# Microtomographic Assessment of the Shaping Ability of the Hyflex CM and XP-endo Shaper Systems in Curved Root Canals

**DOI:** 10.1055/s-0042-1750694

**Published:** 2022-10-11

**Authors:** Wania Christina Figueiredo Dantas, Marilia Fagury Videira Marceliano-Alves, Eduardo Fagury Videira Marceliano, Eduardo Fernandes Marques, Thais Machado de Carvalho Coutinho, Flavio R.F. Alves, Alexandre Sigrist De Martin, Rina Andrea Pelegrine, Ricardo Tadeu Lopes, Carlos Eduardo da Silveira Bueno

**Affiliations:** 1 Department of Endodontics, São Leopoldo Mandic College, São Leopoldo Mandic Research Institute, Campinas, São Paulo, Brazil; 2 Department of Endodontics and Dental Research, Iguaçu University, Nova Iguaçu, Brazil; 3 Department of Dental Prosthesis, Belem General Hospital, Belém, Pará, Brazil; 4 President Antonio Carlos University, Porto Nacional, ITPAC, Porto/ FAPAC, Palmas, Brazil; 5 Departament of Nuclear Energy, Rio de Janeiro Federal University, Rio de Janeiro, Brazil

**Keywords:** root canal therapy, microtomographic analysis, unprepared canal walls, rotary instruments, Hyflex CM, XP-endo Shaper, curved canals

## Abstract

**Objective** This study compared the shaping ability of the Hyflex CM and XP-endo Shaper rotary file systems in curved mesial canals of mandibular molars using micro-computed tomography.

**Material and Methods** Seventeen mesial roots of extracted first mandibular molars with two independent mesial canals were scanned before and after root canal preparation with the tested rotatory file systems. Each mesial canal from the same specimen was prepared with one of the two systems. The parameters analyzed were canal centering (transportation) for the cervical, middle, and apical segments, as well as for the entire canal (0–10 mm from the apex); and canal volume increase, canal surface area increase, and unprepared canal walls for two segments, 0 to 4 mm and 0 to 10 mm from the apex.

**Results** There was no significant difference between both systems regarding canal centering (transportation), volume increase, and unprepared canal walls for the 0 to 10 mm segment ( *p* > 0.05); however, a significant difference was observed for the 0 to 4 mm segment ( *p* <0.01), where the Hyflex CM left 28.46% of unprepared walls and XP-endo Shaper left 13.26%.

**Conclusions** The shaping ability of the two tested rotatory file systems in mesial roots of first mandibular molars was similar for all parameters in all the segments evaluated, except for the 0 to 4 mm segment, where XP-endo Shaper left a smaller area of unprepared canal walls than Hyflex CM.

## Introduction

During preparation of the root canal system, the mechanical action of instruments combined with irrigation facilitates subsequent operative procedures; it promotes canal debridement, creates space for intracanal medication, and optimizes the root canal filling procedure. 1 With the introduction of nickel-titanium (NiTi) rotary systems, endodontic instrumentation has become faster and safer, favoring the preservation of the original canal anatomy. 1 Despite the lower rates of procedural errors—such as zips and perforations—associated with these instruments, a certain amount of dentinal wall area remains unprepared after preparation, possibly causing failures in endodontic treatment. 2 In curved canals, incomplete dentin removal in one portion of the canal, and excessive removal in the other, can increase the risk of apical transportation, as well as fracture and weakening of the root structure. 3 4 Even though the improvements observed in the manufacturing process of NiTi rotary instruments three-dimensional root canal preparation remains a major challenge to endodontists, and instruments with different geometries and surfaces have been introduced to render canal shaping more predictable. 1 2 3

The first memory-controlled rotary instruments were launched in 2010, namely the Hyflex CM system (Coltène/Whaledent, Allstätten, Switzerland). This controlled-memory effect is achieved through a special thermomechanical treatment. The system has multiple instruments with a triangular cross-section (sizes 25/.08 and 20/.06), and a quadrangular cross-section (sizes 20/.04, 25/.04, 30/.04, and 40/.04). The flexural strength of its instruments is up to 300% higher than that of instruments made with conventional NiTi alloys, 5 6 7 enabled by the predominance of the martensitic phase in its alloy. 8 9

The XP-endo Shaper instrument (FKG, La Chaux-de-Fonds, Switzerland) was introduced in 2016 following the single-file preparation concept and is made from the MaxWire alloy (Martensite-Austenite Electropolishing Flex, FKG). This alloy undergoes a martensitic-to-austenitic phase transformation with an increase in temperature (35°C), thereby promoting expansion of the instrument from its original 30/.01 size to a new 30/.04 size. This means that expansion of the preparation takes place in a single step, without requiring the use of successively larger files. 10 11 12 13

Microtomographic (micro-CT) analysis has been widely used in endodontic research because it is a non-destructive method with excellent accuracy and is considered the gold standard for assessing the three-dimensional shaping ability of endodontic instrumentation systems. 14 15 16

It is important to study new rotary systems such as Hyflex CM and XP-endo Shaper, as they present unique design and provide superior flexibility, thus allowing better maintenance of the original canal curvature and greater efficiency and safety. 14 15

Even after conducting an extensive literature review on the subject, no study was found comparing a memory-controlled NiTi alloy multiple-file rotary system versus a MaxWire NiTi alloy single-file rotary system with respect to root canal shaping in teeth with curved roots. Thus, the aim of the present study was to assess the shaping of curved mesial canals of mandibular molars produced with the Hyflex CM or XP-endo Shaper systems by a micro-CT analysis of preparation centering (apical transportation), canal volume, canal surface area, and unprepared dentin wall parameters. The null hypothesis tested was that both systems would provide equivalent canal shaping with respect to the parameters analyzed.

## Materials and Methods

### Sample Size Calculation

Sample size was calculated using the Cohen method (1988). Considering test power of 80%, a sampling error of 5%, and an effect size of 0.85, it was determined that a minimum number of 17 specimens per study group were required.

### Selection of Specimens

This study was approved by the local institutional research ethics committee (register no. 2.270.631). The specimens used were teeth indicated for extraction for reasons unrelated to this research and were donated by patients to one of the authors. After extraction, they were kept in distilled water in the institutional bio-repository for a maximum period of 3 months.

From pool of 102 mandibular molars, those that met the following criteria were selected: intact roots, complete root formation, no previous endodontic treatment, and severe curvature—between 20 and 30 degrees (Schneider 1971). In addition, the mesial roots had to have two independent foramina (Vertucci type IV configuration), whose existence was confirmed by introducing a n. 10 K-type file (Dentsply Maillefer, Ballaigues, Switzerland) in either canal until it was seen exiting the respective apical foramen under the lens of an operating microscope (Alliance 20x, Alliance Comercial de São Carlos, São Carlos, SP, Brazil). Thus, 54 mandibular molars were selected and included in the sample.

The roots were scanned using a SkyScan 1173 micro-CT apparatus (Bruker Micro-CT, Kontich, Belgium) set to operate at 50 kV, 80 µA, 360 degrees rotation around the vertical axis with a rotation step of 0.9 degrees and using a 1-mm thick aluminum filter and a pixel size of 12.11 µm. Once obtained, the images were reconstructed from cross sections using NRecon v1.6.9.0 software (Bruker Micro-CT). The values of the length, volume, surface area, and structure model index parameters of the canals were obtained using CTAn v.1.14.4 software (Bruker Micro-CT). Seventeen mesial roots of mandibular molars with similar values ( *p* > 0.05) for these parameters were finally included in the study. Similarly, the mesial canals were matched based on their similarity in terms of micro-CT measurements, and then randomly distributed ( www.random.org ) into the two experimental groups.

Seventeen mesial roots of mandibular molars with similar values ( *p* > 0.05) for these parameters were eventually included in the study. Likewise, the mesial canals were matched based on their similarity in micro-CT measurements and randomly assigned ( www.random.org ) to the two experimental groups.

### Root Canal Preparation

All the experimental procedures were performed by a single operator, who was experienced in the tested systems. The instruments were used only once, as a deformity in the instrument could occur without any visible warning sign. The working length (WL) was established at 1 mm short of the apical foramen. The root apex was sealed with gingival isolation material (Topdam; FGM, Joinville, Santa Catarina, Brazil) to create a closed system. Preparation was performed with the Hyflex CM and XP-endo Shaper systems alternately in each mesial canal, to minimize possible influence of anatomical variation between specimens. Glide-path creation and maintenance of foraminal and canal patency throughout the instrumentation procedure were performed in all the canals with a n. 15 K-type file, up to 1 mm short of the actual tooth length, and with a n. 10 K-type file, up to 1 mm beyond this length, respectively.

The procedures were performed inside a booth containing a heater (800-Heater; PlasLabs, Lansing, Michigan, United States) that maintained an ambient temperature of 37°C to simulate actual clinical conditions.

### Hyflex CM (Group HFCM)

Hyflex CM instruments were driven by a VDW Silver electric motor (VDW, Munich, Germany), operating at a speed of 500 rpm and a torque of 2.5 N.cm. The canals were instrumented using the crown-down technique, starting with a 25/.08 instrument introduced up to the first two-thirds of the canal, followed by the 20/.04, 25/.04, 20/.06, and 30/.04 instruments, taken up to the WL.

### XP-endo Shaper (Group XPS)

The XP-endo Shaper instrument was driven by a VDW Silver electric motor (VDW), operating at a speed of 800 rpm and a torque of 1.0 N.cm. Once the instrument was introduced into the canal, two gentle in-and-out movements toward the apex and up to the WL were performed. After five repetitions of this instrumentation sequence, canal shaping was tested with a 30/.04 gutta-percha cone (VDW), at which time it was considered complete.

Before beginning instrumentation, the pulp chamber of the specimens from both groups was filled with 2 mL of a 2.5% sodium hypochlorite (NaOCl) solution.

During instrumentation, canal irrigation was performed with 2 mL of 2.5% NaOCl at each instrument change, in Group HFCM, and after each in-and-out movement, in Group XPS, totaling 10 mL of solution per canal. A disposable hypodermic syringe attached to a NaviTip 30-G irrigation needle and positioned 2 mm short of the WL was used in the procedure.

Final irrigation in both groups was performed with a 5 mL of 17% ethylenediaminetetraacetic acid (EDTA), followed by 5 mL of 2.5% NaOCl; the canals were dried with absorbent paper points (Dentsply-Maillefer).

### Micro-CT Evaluation

**Fig. 1:**
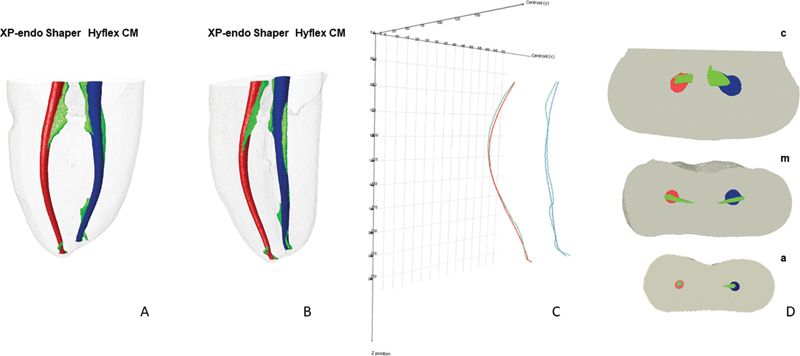
( **A** – **D** ) Representative of final microtomographic image after preparation of mesial canals with the Hyflex CM ( *in blue* ) and XP-endo Shaper ( *in red* ) systems; the unprepared dentin walls are represented in green.

After root canal preparation, a postoperative scan was performed, and the images were reconstructed following the same protocol used in the initial scan. The model images were color-coded to allow a qualitative comparison of the canal shaping produced by the Hyflex CM and XP-endo Shaper systems; the initial scans were coded in green, and the final scans—referring to the results obtained after using either system—in blue or red, respectively ( Fig. 1 ).

CTAn v.1.16.4.1 software (Bruker micro-CT) was used to measure the volume and surface area of the canals. Pre- and postoperative images were superimposed using Image J v. 1.50d software (National Institute of Health, Bethesda, Maryland, United States) to determine the value of the parameter of the unprepared surface area of the canal. The area of this surface was determined by computing the static voxels (those voxels whose position on the canal surface was unchanged after preparation) and expressed as a percentage.

Canal centering ability was evaluated based on data on the variation of the centers of gravity for each canal segment, connected along the Z axis, and the mean transportation observed in each root segment (in mm) was calculated by comparing the centers of gravity before and after preparation in the respective segments. Similarly, the transportation for the entire canal was determined by averaging the three segments in each specimen.

### Statistical Analysis

Data distribution was analyzed using the Shapiro-Wilk normality test. The Wilcoxon test was used to perform intragroup comparisons between the two evaluated canal segments, 0 to 10 mm and 0 to 4 mm from the apex, with respect to the canal volume and surface area parameters. Friedman's test was used to perform intragroup comparisons among the cervical, middle, and apical segments for the center of gravity parameter (canal transportation). The Mann-Whitney test was used to perform intergroup comparisons for pre-preparation values of canal volume, and post-preparation values of canal volume increase, surface area increase (%), unprepared areas, and canal transportation in the cervical and middle segments. The independent *t* -test was used to perform intergroup comparisons for pre-preparation surface area, and post-preparation percentage of unprepared areas, in the 0 to 10 mm and 0 to 4 mm segments, and for variation in center of gravity in the apical segment and in the entire canal. All the statistical tests were performed using a level of significance of 5% ( *p* <0.05).

## Results

### Canal Volume, Surface Area, and Unprepared Surface Area

Root canal volume values are shown in Table 1 . Surface area and unprepared surface area, before and after preparation, are shown in Table 2 . Initial canal volume and surface area were similar in both groups ( *p* >0.05) and increased significantly after preparation with both the systems tested ( *p* <0.01). There was no significant difference between the groups with respect to the percentages of volume increase in the entire canal, and with respect to unprepared areas in the 0 to 10 mm segment ( *p* >0.05; Table 2 ). However, a significant difference was observed with respect to unprepared areas in the 0 to 4 mm segment, with HFCM specimens showing significantly higher percentages of unprepared areas than XPS specimens ( *p* <0.01).

**Table 1:** Root canal volume (mm ^3^ ) along the entire length of the canal (from 0 10 mm from the apex), before and after preparation performed with the systems tested in the study

Group	Segment		Mean (SD)	Median	Interval
		Initial	114,915.29 (21,683.11)	105,147	85,411–166,519
	**0–10 mm**	Final	17,627.14 (5,449.96)	17,012	8,555–27,369
		**% unprepared**	** 15.81 ^A^ (5.25) **	16.37	5.67–24.26
**HFCM**					
		Initial	20,695 (8,132.84)	22,016.50	7,206–31,637
	**0–4 mm**	Final	6,021.28 (2,874.96)	6,093.50	1,917–10,269
		**% unprepared**	** 28.46 ^B^ (4.48) **	27.80	21.67–36.75
		Initial	119,266.57 (46,491.43)	109,767.50	49,163–197,619
	**0–10 mm**	Final	15,047.86 (9131.30)	15,807	3,721–31,138
**XPS**		**% unprepared**	** 12.89 ^A^ (6.62) **	13.36	3.32–23.44
		Initial	25,588.43 (12,012.82)	23,176.50	9,554–51,912
	**0–4 mm**	Final	3,170.64 (1671.38)	2,516.50	1,059–6,435
		**% unprepared**	** 13.26 ^A^ (4.82) **	13.29	3.58–19.52

* Values followed by the same superscript letter within columns indicate a statistically significant difference.

**Table 2:** Untouched root canal surface area (in number of static voxels) in the two segments evaluated (0–4 mm and 0–10 mm from the apex), before and after preparation performed with the systems tested in the study

Group	Segment	Mean (SD)	Median	Interval
	Cervical	** 0.75 (0.38) ^A^**	0.92	0.05–1.15
	Middle	** 0.52 (0.29) ^A^**	0.53	0.05–0.98
HFCM				
	Apical\	** 0.35 (0.21) ^B^**	0.31	0.03–0.69
	0–10 mm	0.33 (0.15)	0.33	0.09–0.58
	Cervical	0.56 (0.36)	0.50	0.02–1.07
	Middle	0.46 (0.39)	0.52	0.02–0.98
XPS				
	Apical	0.36 (0.17)	0.35	0.08–0.60
	0–10 mm	0.49 (0.26)	0.57	0.01–0.79

a Values followed by the same superscript letter within columns indicate a statistically significant difference.

### Canal Centering Ability

In general, there was no variation between the groups or among the segments within the same group with respect to the center of gravity parameter (canal transportation; *p* >0.05); The only exception was found in the HFCM intragroup analysis, which revealed the occurrence of significantly less canal transportation in the apical segment than in the cervical and middle segments ( *p* <0.05).

## Discussion

The aim of the study was to conduct a microtomographic comparison of two rotary instrumentation systems—the single-file XP-endo Shaper and the multiple-file Hyflex CM—with respect to their shaping ability in mesial root canals of mandibular molars. The volume and surface area of the canal, the percentage of unprepared walls, and the centering of the preparation were evaluated. The null hypothesis was partially accepted, since there were statistically significant intergroup and intragroup differences with respect to some of the parameters evaluated.

Root canal anatomy has a direct influence on the quality of the preparation produced by endodontic instrumentation. Mandibular molars were used in the present study because these teeth are most indicated for endodontic treatment. 17 Their roots have moderate to severe curvature, with a higher prevalence of curvature in their mesial canals. 6 14

This factor increases the risk of apical transportation during instrumentation. 18 19 In addition, mandibular molars often have independent root canals in the mesial root, 20 this enabled specimen standardization between the groups in the present study, since each of the two root canals of the same root was instrumented using either of the systems tested, thus mitigating possible interference of anatomical variation in the results. Furthermore, all the procedures were performed by the same operator in a booth heated to 37°C, considering that instruments submitted to heat treatment during their manufacturing process may present a structural phase change at higher temperatures. 10

Micro-CT was selected as the method of analysis, because it is a widely used, non-destructive method that provides a 3D evaluation of the instrumentation results, without requiring any alteration of root anatomy. As a result, it is considered the gold standard for this type of analysis 14 to 16.

The XP-endo Shaper system consists of a new generation of instruments that have the ability to expand beyond their nominal size, thus touching a larger area of dentin walls during preparation and removing a greater amount of debris. 21 In the present study, its shaping ability was compared with that of the Hyflex CM system, which is composed of controlled-memory- NiTi instruments. Owing to their high flexibility and heat treatment, Hyflex CM instruments are suitable for the instrumentation of curved canals and have shown a canal-centering ability superior to that of conventional NiTi files. 22 However, unlike the XP-endo Shaper, these instruments lack the ability to contract or expand beyond their core to better adapt to root canal anatomy. 12

In the present study, specimens from Group XPS were associated with a lower percentage of unprepared walls in the apical segment; however, their walls were not completely instrumented, corroborating the results of previous studies. 10 23 The XP-endo Shaper instrument is made from MaxWire alloy (Martensite-Austenite Electropolishing-Flex, FKG), which imparts a more rectilinear shape to the instrument when cooled (martensitic phase), and then a “snake-like” shape when submitted to body temperature (austenitic phase). This expandability, combined to its small mass, seems to have contributed to the three-dimensional shaping of the canal, especially in the apical third. In addition, owing to this change in crystal structure and to its 6-edge “booster tip,” the XP-endo Shaper instrument can begin instrumentation following a glide-path with an n. 15 diameter, and then expand to an n. 30 diameter. Previous research has found that 17, 10 31, 24 and 9.42% 12 of the total root canal wall area remained unprepared by the XP-endo Shaper file, whereas 13% of the canal walls were found to be unprepared in the present study. This variation in results could be explained by differences among specimens with respect to root canal anatomy. 25

A significant difference was observed in relation to unprepared areas in the 0 to 4 mm segment ( *p* <0.01), with specimens from the HFCM Group showing significantly higher percentages of unprepared areas than those from the XPS group. Other authors reported that the fewer the areas a file works in root canal system, the greater the remaining pulp tissue and microorganisms that may persist, thus contributing to reinfection as well as interfering with obturation. 15 26

Inadvertent apical transportation during root canal preparation depends on the degree and radius of canal curvature, and, even more so, on the choice of instruments. 22 In our study, no significant difference was found between the instruments regarding this parameter, demonstrating that there was little variation between them with respect to their ability to produce well-centered preparations. This result could be explained by the high flexibility of both systems; furthermore, it suggests that the type of alloy used in the instruments may be partially responsible for their mechanical behavior in curved canals, 6 19 24 27 28

Intragroup analysis revealed that the Hyflex CM system promoted a lower degree of transportation in the apical third than in the cervical third. Although the instrument used for apical preparation in this group was the 30/.04, the one used for cervical preparation was the 25/.08, which has a triangular cross-section. 25 Although not specifically designed to act as a canal orifice enlargement file (or “orifice shaper”), this 25/.08 instrument promoted an enlargement corresponding to its size in the cervical region, which may have contributed to obtaining this lower transportation result.

This aspect is noteworthy, because a substantial enlargement of the cervical third can lead to root weakening due to widening of the walls facing the furcation region. 4 29 With respect to the apical third, any transportation above 0.3 mm may have a negative impact on treatment success rates. 29 30 A previous study found low percentages of apical transportation for the Hyflex CM system in the apical and cervical thirds 6 ; however, it should be borne in mind that the preparation in their study was performed only up to an n. 25 instrument.

The concept of employing a single NiTi instrument to prepare the entire root canal 31 has been proven to enable a shorter learning curve and provide effective preparation. 12 13 32 In the present study, the single-file XP-endo Shaper system displayed a shaping ability similar to that of a multiple-file Hyflex CM system; in addition, it prepared a larger area of dentinal walls in the apical segment. The XP-endo Shaper was used following manufacturer's recommendations, according to which the preparation can be considered complete after five movements toward the WL, followed by another five movements if needed, totaling 10 instrument penetrations; however, other study demonstrated that an increase in instrumentation time with this system led to an increase in the percentage of prepared walls, canal volume, and dentin removal, suggesting a correlation between preparation quality and instrument application time. 8 9 28

Further research is warranted to confirm the indication for using a single-file system, and to explore aspects still lacking investigation in the literature, including possible adjustments to the protocols of use and the development of new treatments for NiTi alloys.
